# Biofilm Medium Chemistry and Calcium Oxalate Morphogenesis

**DOI:** 10.3390/molecules26165030

**Published:** 2021-08-19

**Authors:** Aleksei Rusakov, Maria Kuz’mina, Olga Frank-Kamenetskaya

**Affiliations:** Crystallography Department, Institute of Earth Sciences, St. Petersburg State University, Universitetskaya nab. 7/9, 199034 St. Petersburg, Russia; m.kuzmina@spbu.ru

**Keywords:** microbe biomineralization, calcium oxalate crystallization, weddellite, whewellite

## Abstract

The present study is focused on the effect of biofilm medium chemistry on oxalate crystallization and contributes to the study of the patterns of microbial biomineralization and the development of nature-like technologies, using the metabolism of microscopic fungi. Calcium oxalates (weddellite and whewellite in different ratios) were synthesized by chemical precipitation in a weakly acidic environment (pH = 4–6), as is typical for the stationary phase of micromycetes growth, with a ratio of Ca^2+^/C_2_O_4_^2−^ = 4.0–5.5, at room temperature. Additives, which are common for biofilms on the surface of stone in an urban environment (citric, malic, succinic and fumaric acids; and K^+^, Mg^2+^, Fe^3+^, Sr^2+^, SO_4_^2+^, PO_4_^3+^ and CO_3_^2+^ ions), were added to the solutions. The resulting precipitates were studied via X-ray powder diffraction (XRPD), scanning electron microscopy (SEM) and energy dispersive X-ray spectroscopy (EDXS). It was revealed that organic acids, excreted by micromicetes, and some environmental ions, as well as their combinations, significantly affect the weddellite/whewellite ratio and the morphology of their phases (including the appearance of tetragonal prism faces of weddellite). The strongest unique effect leading to intensive crystallization of weddellite was only caused by the presence of citric acid additive in the medium. Minor changes in the composition of the additive components can lead to significant changes in the weddellite/whewellite ratio. The effect of the combination of additives on this ratio does not obey the law of additivity. The content of weddellite in the systems containing a representative set of both organic acids and environmental ions is ~20 wt%, which is in good agreement with natural systems.

## 1. Introduction

In recent years, the scientific community from all over the world has shown a significant interest in the mechanisms of biomineralization with the participation of microorganisms, which is associated with the study of modern mineral formation at nano- and microlevels, as well as processes and phenomena occurring at the border of living and non-living [1,2,3]. The study of microbial biomineralization also creates a scientific basis for the development of modern nature-like biotechnologies (ore enrichment; monuments conservation, primarily cracks healing; environment bioremediation, etc.) [3,4,5].

The present study is devoted to oxalate crystallization under the action of microorganisms. Oxalic acid salts (oxalates) are usually localized in biofilms with dominance of lichens and microscopic fungi, which are located on the surface of rocks and minerals [6]. Active microbe metabolism products which contain organic acids (including oxalic) dissolve the underlying substrate and initiate oxalate crystallization. Cations involved in their formation enter the biofilm from the underlying stone substrate, and the environment (atmospheric air and soil) [7]. Acid rains, dews and fogs containing aerosols of various ions (including heavy metals) strongly contribute to oxalate crystallization.

Calcium oxalates (monohydrous whewellite CaC_2_O_4_·H_2_O and dihydrous weddellite CaC_2_O_4_·(2 + x)·H_2_O) are the most common biofilm minerals [3]. They were found in lichen thalli on the surface of different calcium-containing rocks and minerals [8,9,10,11,12], as well as on artificial items (paintings, writing materials and even mummified human remains) [13,14]. Calcium oxalates under the action of microscopic fungi on the surface of rocks and minerals were also obtained in model experiments [3,15,16,17,18,19,20].

Whewellite and weddellite stability in temperate environmental conditions have previously been investigated, and it was shown that whewellite is the more stable of the two [18]. It was also discovered that the stability of weddellite depends on the content of water molecules in crystal structure [18,21,22].

The results of works on reasons for the ubiquitous presence of unstable weddellite, as well as the factors controlling its formation, are contradictory [4]. Oxalate patina studies showed that, under 20 °C, weddellite precipitates under excess of calcium ions in the medium, while whewellite precipitates under a higher concentration of oxalate ions. Weddellite also appears to be favored by low temperatures and a very humid microenvironment with very little exposure to sunlight [12]. Model experiments confirmed that whewellite is stable above room temperature and that weddellite naturally transforms into whewellite via a one-step dehydration process [17]. The stability of whewellite is favored by dry conditions, and the mechanism of dehydrate-to-monohydrate transformation is not the only source of whewellite [18].

In our previous experimental work, it was established that fungal biofilm components may facilitate the formation of metastable calcium oxalate weddellite in crystallization range of whewellite and affect its crystal habit [23]. The decisive role in the formation of weddellite crystals at temperatures near 20 °C, according to our data, is played by the ratio between Ca^2+^ and oxalate ions. The citric acid also released by micromycetes, along with the oxalic acid and Fe^3+^ ions entrapped by biofilm from the environment, plays a significant role in weddellite precipitation.

This work is a continuation of our previous researches. The main goal was to expand the set of biofilm components (organic acids and environmental ions) and establish the influence of medium chemistry on calcium oxalates’ formation and their morphology.

## 2. Results

### 2.1. The Precipitate Phase Composition and the Morphology of Calcium Oxalate Crystals

**Figure 1:**
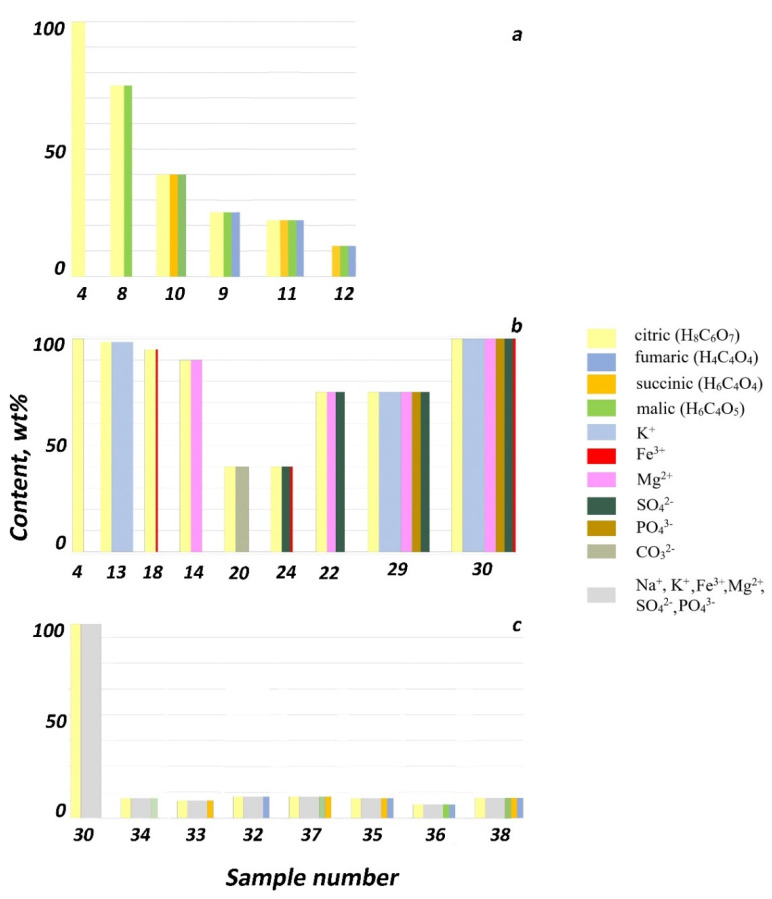
The content of weddellite in precipitate (obtained via XRPD) in the presence of different additives in solution: (**a**) organic acids, (**b**) citric acid and environmental ions and (**c**) organic acids and environmental ions. Sample numbers correspond to Appendix A Table A1 and Table A2. Pillar thickness corresponds to the relative concentration of the additive in the solution. The mixture of environmental ions in (**c**) is grayed for the ease of diagram reading.

**Figure 2:**
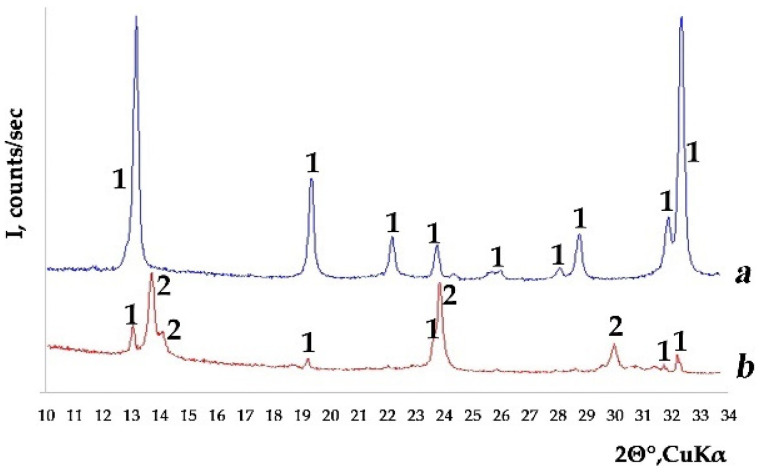
Typical XRPD patterns of the studied samples: (**a**) weddellite in Synthesis 30 and (**b**) whewellite and weddellite mixture in Synthesis 33. Designation: weddellite (1) and whewellite (2).

Calcium oxalates were obtained from solutions of various compositions (Ca^2+^/C_2_O_4_^2−^ = 4–5) in all syntheses pH_init_ = 4–8 (Appendix A Table A1): whewellite (Syntheses 1 and 2; Appendix A Table A2) and weddellite (Syntheses 4–7, 17–19 and 30; Appendix A Table A2). However, most often, a mixture of whewellite and weddellite in various ratios (all other syntheses; Appendix A Table A2; Figure 1 and Figure 2). Weddellite crystal (and their intergrowth) sizes varied in different syntheses, from 0.6 to 100–200 µm, but were mainly in the range of 4–30 µm. Whewellite crystal sizes varied in the range from 2 to 40 µm. The change in the pH value during the formation of calcium oxalates (pH_fin_–pH_init_ up to 1.9; Appendix A Table A1) depends on the composition of the solution. The maximum increase occurred in a solution of a complex composition containing citric acid and the following environmental ions: K^+^, Mg^2+^, SO_4_^2−^ and PO_4_^3−^ (Synthesis 27).

#### 2.1.1. Syntheses without Additives

**Figure 3:**
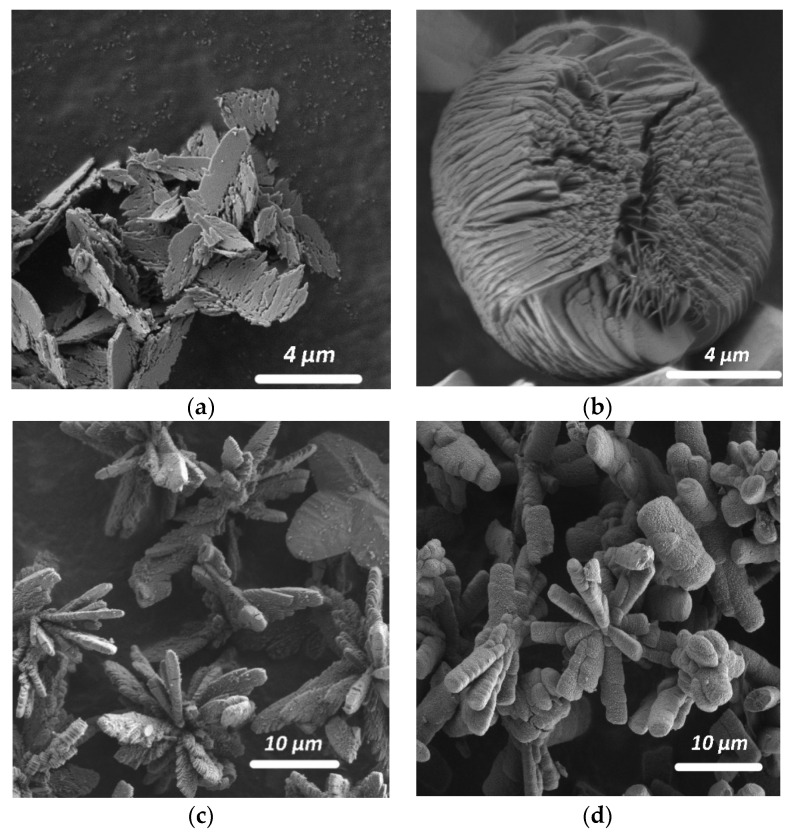
Morphology of whewellite crystals and their intergrowths synthesized from solutions of various composition: (**a**) without additional additives (Synthesis 1); (**b**) with the addition of K^+^, Mg^2+^, SO_4_^2−^ ions and citric acid (Synthesis 29); (**c**) with the addition of K^+^, Mg^2+^, Fe^3+^, SO_4_^2−^, PO_4_^3−^ ions (Synthesis 3); and (**d**) with citric and fumaric acids and K^+^, Mg^2+^, Fe^3+^, SO_4_^2−^, PO_4_^3−^ ions (Synthesis 32).

In the absence of any additives, the obtained precipitate consisted only of whewellite (Synthesis 1), which formed as poorly faceted elongated lamellar crystals (~7 μm in size) and their intergrowths (Figure 3a).

#### 2.1.2. Syntheses with the Addition of Environmental Ions

The presence of any environmental ion or their combination in the solution in the absence of additives of organic acids secreted by microscopic fungi (except for obviously oxalic) practically did not change the phase composition of the precipitate, which was represented by only whewellite (Synthesis 2) or whewellite with a very small amount weddellite (Synthesis 3). With the addition of K^+^, Mg^2+^ and SO_4_^2^^−^ ions to the solution (Synthesis 2), whewellite crystals formed as spherulites (Figure 3b); with the higher amount of environmental ions added to the crystallization medium (K^+^, Mg^2+^, Fe^3+^, SO_4_^2^^−^ and PO_4_^3^^−^ ions), large volumetric radiant intergrowths (~17 μm in size) composed of small lamellar crystals of whewellite appeared (Figure 3c). Small dipyramidal crystals of weddellite (15 μm in size) were visible among whewellite intergrowths.

#### 2.1.3. Syntheses with the Addition of Organic Acids

**Figure 4:**
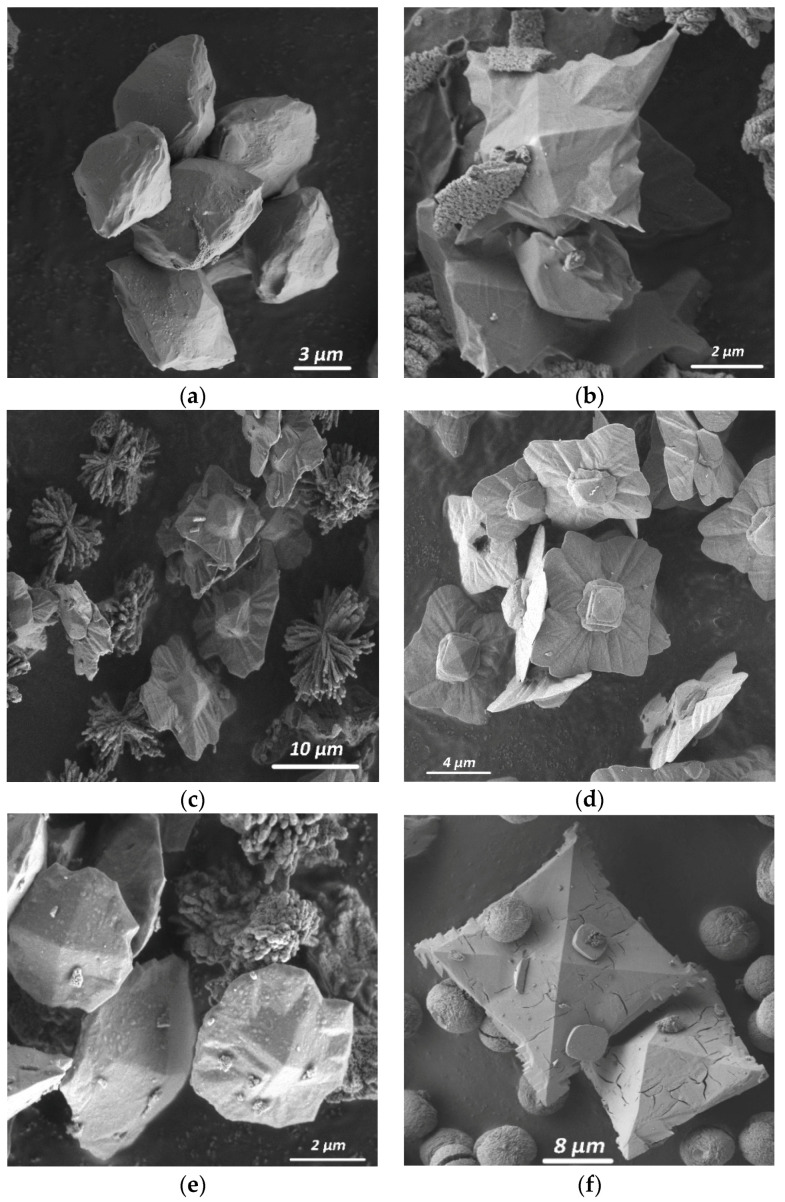

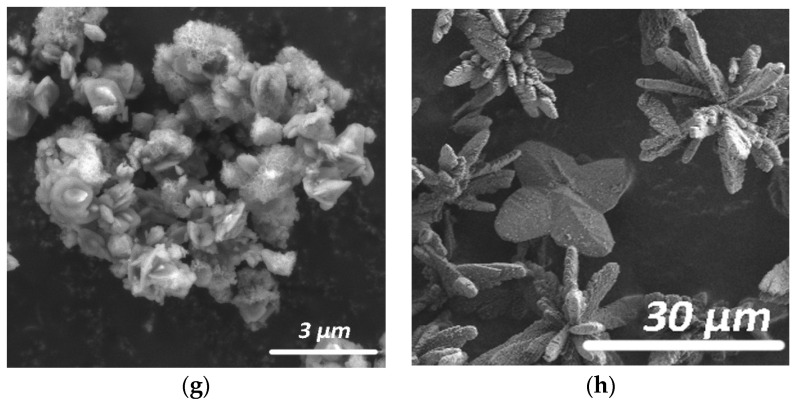
Morphology of weddellite crystals synthesized in the presence of various additives: (**a**) with citric acid (Synthesis 5); (**b**) with malic, fumaric and succinic acids (Synthesis 12); (**c**) with citric acid and Mg^2+^ cations (Synthesis 15); (**d**) with citric acid and Fe^3+^ cations (Synthesis 17); (**e**) with citric acid and Fe^3+^ and SO_4_^2−^ ions (Synthesis 24); (**f**) with citric acid and carbonate ions (Synthesis 20); and (**g**,**h**) with citric acid and K^+^, Mg^2+^, Fe^3+^, SO_4_^2−^, PO_4_^2−^ ions (Synthesis 30).

The addition of citric acid contributed to the formation of almost monophasic weddellite precipitates (Appendix A Table A2, Syntheses 4–7; Figure 1a). Weddellite crystals had the dipyramidal faceting (6 μm in size, Figure 4a). Few whewellite crystals (15–30 μm in size) formed as spherulites, just as in Synthesis 29 (Figure 3b).

Mixing citric acid with other organic acids secreted by micromycetes (succinic, fumaric or malic), one by one or in combination with each other, led to a decrease in the amount of weddellite in the precipitate (Syntheses 8 and 11). The more additional acids were present, the less weddellite formed (Figure 1a).

As shown by us earlier, in the absence of citric acid, none of these acids added to the solution solely led to the formation of weddellite [23]. In the case of the simultaneous presence of succinic, fumaric and malic acids in the crystallization medium in a small and approximately equal amounts, weddellite precipitated in small amount (Synthesis 12, Figure 1a) as flat dipyramidal crystals (5 μm in size, Figure 4b).

#### 2.1.4. Syntheses with the Addition of Citric Acid + Environmental Ions

When synthesizing calcium oxalates with the addition of citrate ions and various environmental ions in different ratios, weddellite and whewellite formed in different ratios to each other (Appendix A Table A2, Syntheses 13–31; Figure 1b).

The separate addition of several ions (K^+^, Mg^2+^, Fe^3+^ and CO_3_^2−^) to citric acid led to a variable decrease of weddellite amount. The presence of K^+^ (Synthesis 13), Fe^3+^ (Synthesis 18) and Mg^2+^ (Synthesis 14) led to a slight decrease of weddellite amount (Syntheses 16–19). The addition of CO_3_^2−^ ions (Synthesis 20) resulted in the least amount of weddellite formed.

**Figure 5:**
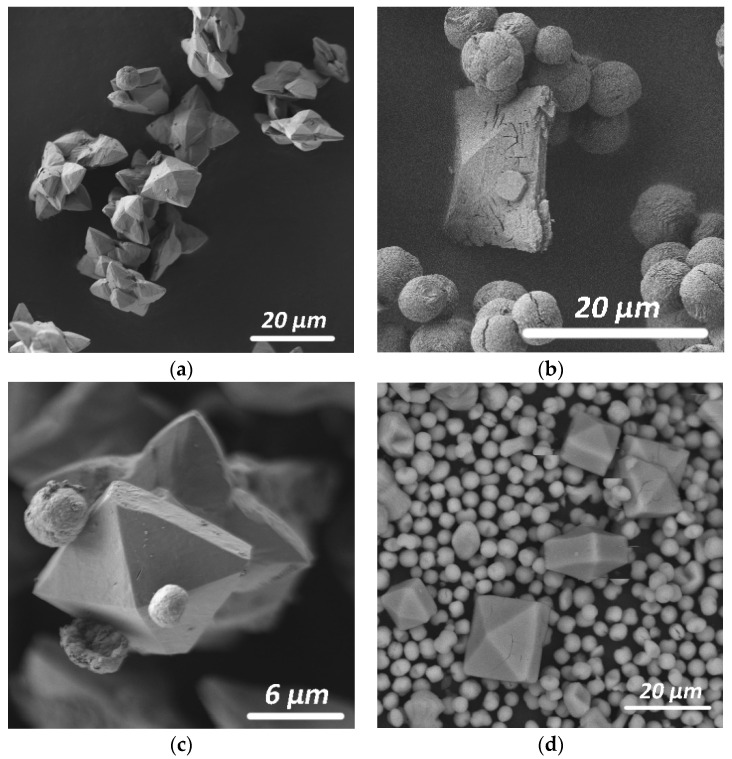
Tetragonal prism faces in the faceting of weddellite crystals synthesized with citrate ions and some environmental ions: (**a**) with potassium cations (Synthesis 13), (**b**) with magnesium and sulfate ions (Synthesis 23), (**c**) with carbonate ions (Synthesis 20) and (**d**) with strontium cations [24].

The addition of K^+^ cations (Synthesis 13) promoted the appearance of small prism faces on the crystals (Figure 5a). When CO_3_^2−^ ions had been added (Synthesis 20), smaller faces of a tetragonal prism appeared (Figure 5b). In addition, rather large flat quadrangular weddellite plates with sharp serrated corners formed (5 μm in size, Figure 4e). With the addition of Fe^3+^ ions, weddellite appeared as skeletal crystals: square plates with a wavy surface and a tetragonal dipyramid in the center of each plate (15 μm in size, Figure 4d).

The addition of Mg^2+^ together with CO_3_^2−^ ions led to the appearance of a large amount of weddellite, comparable to the synthesis with Mg^2+^ ions (Synthesis 22). With the addition of SO_4_^2−^ and Mg^2+^ cations, as well as SO_4_^2−^ or PO_4_^3−^ anions, together with Fe^3+^, led to a very small amount of weddellite (Syntheses 22, 24 and 31)—the same as in the case of the addition of carbonate ion (Figure 1b). Those weddellite crystals had the shape of slightly flattened dipyramids with poorly defined, rounded edges (20 μm in size, Figure 4d). The presence of magnesium and sulfate ions (Synthesis 23) almost always ensured the formation of prism faces, as well (Figure 5c).

The simultaneous addition of multiple components (Syntheses 27–31) also leads to mixed results. When multiple environmental ions (K^+^, Mg^2+^, PO_4_^3−^ or K^+^, Mg^2+^, SO_4_^2−^, PO_4_^3−^ or K^+^, Mg^2+^, Fe^3+^, SO_4_^2−^, PO_4_^3−^), together with citric acid, were added to the medium, the content of weddellite was significantly higher than that of whewellite (Syntheses 27–98 wt%, Syntheses 29–89 wt% and Syntheses 30–100 wt%), which is less than or equal to the amount of weddellite in the synthesis with the addition of citric acid only (Synthesis 4). With the addition of other environmental ions combinations (K^+^, Sr^2+^ and PO_4_^3^ in Synthesis 28; and K^+^, Mg^2+^, Fe^2+^, SO_4_^2−^, PO_4_^3−^ and CO_3_^2−^ in Synthesis 31), less weddellite formed compared to whewellite (22 wt% for Synthesis 28 and 9 wt% for Synthesis 31), while, in Synthesis 31, the amount of weddellite was minimal. The bulk of weddellite crystals were represented by flattened dipyramidal skeletal crystals—the same as in Synthesis 12 (Figure 4b)—while, in Synthesis 31, weddellite crystals had the shape of slightly flattened dipyramids with poorly defined rounded edges that were similar to the ones in Synthesis 22, 24 or 31 (Figure 4e). It is worth noting that, in the presence of Sr^2+^ cations and PO_4_^3−^ ions (as the present study showed, Synthesis 28), we also observed weddellite crystallized with well-developed faces of the tetragonal prism (Figure 5d) which were larger than in the above-described cases.

Whewellite crystals formed in this experimental section (in the presence of environmental ions and various organic acids) formed voluminous branched kidney-shaped aggregates (10 μm in size), consisting of intergrowths of small lamellar crystals (Figure 3d).

#### 2.1.5. Syntheses with Citric Acid; Addition of Organic Acids and K^+^, Mg^2+^, Fe^3+^, SO_4_^2−^ and PO_4_^3−^ Ions

Crystallization from the solution with variable organic acid composition and a constant set of environmental additives led to a drastic decrease in the amount of weddellite (9–23 wt%), compared to the synthesis with the addition of citric acid only and the same set of environmental additives (Syntheses 32–38; Appendix A Table A2 and Figure 1c). The least amount of weddellite was observed in the synthesis with the simultaneous addition of citric, malic and fumaric acids (9 wt% in Synthesis 36).

**Figure 6:**
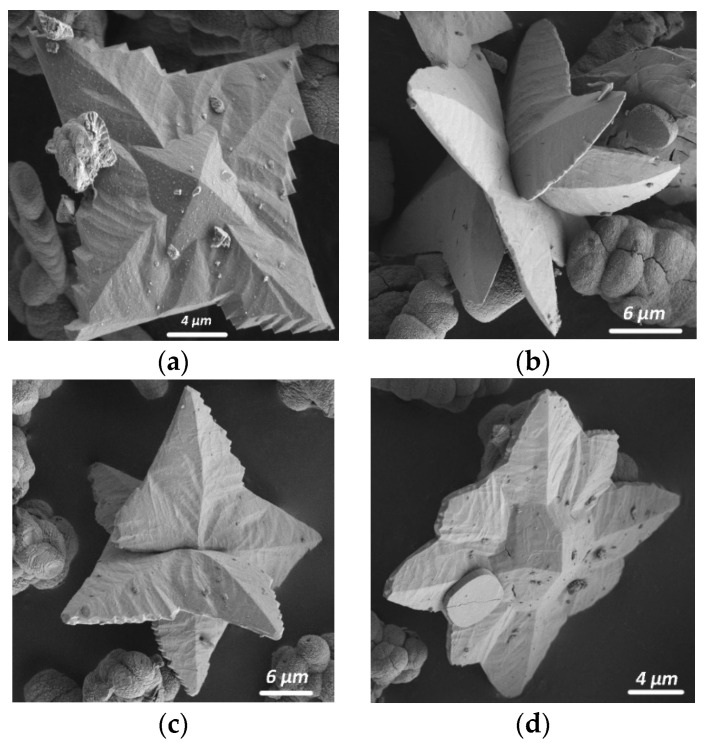
Weddellite crystals and aggregates obtained from solutions with citrate ions, as well as a number of other organic acids and environmental impurities: (**a**) quadrangular flat crystal with a small dipyramid in the center of the plate (Synthesis 33); (**b**,**c**) intergrowths of two or three flattened dipyramidal weddellite crystals along a common plane of symmetry (Syntheses 38 and 35); and (**d**) eight-fold twins, formed by two tetragonal plates intergrowing around a common axis of symmetry of the fourth order and deployed around it by 45° relative to each other (Synthesis 37).

These combinations of additives led to the formation of typical weddellite crystals: quadrangular flat crystals with a small dipyramid in the center of the plate (Syntheses 33, 34 and 36; Figure 6a), as well as crystals with specific morphology, and in particular, various twin intergrowths of weddellite (Figure 6b,c). Weddellite crystals of typical morphology formed in the presence of malic and fumaric acids (20 μm in size). Weddellite crystals of specific morphology formed in the presence of succinic and malic acids (Synthesis 35) or fumaric (Synthesis 38); they were represented by eight-fold twins, formed by two tetragonal plates intergrowing around a common axis of symmetry of the fourth order and deployed around it by 45° relative to each other (40 μm in size, Figure 6d). In the presence of fumaric and succinic acids (Synthesis 35) or fumaric, malic, and succinic acids (Synthesis 38), numerous intergrowths of two or three flattened dipyramidal weddellite crystals were observed along a common plane of symmetry (Figure 6b,c).

Whewellite formed voluminous branched kidney-shaped aggregates (10–25 μm in size), consisting of intergrowths of small lamellar crystals (Figure 3d) similar to the syntheses described in Section 2.1.4.

### 2.2. Crystal Chemistry Characterization of Synthesized Weddellites

The content of most environmental cations (K^+^, Na^+^ and Mg^2+^) in oxalate precipitates did not exceed 1 wt%; Sr^2+^ and Fe^3+^—2 wt% (Appendix A Table A3). The content of S, P and Cl did not exceed 3 wt%.

The unit cell parameters of weddellites synthesized from solutions of various compositions varied significantly (Appendix A Table A2): ***a***—from 12.312 to 12.383 Å, and ***c***—from 7.343 to 7.362 Å. The minimum of unit cell parameters was recorded for weddellite synthesized at a very high pH, i.e., pH = 8.6 (Synthesis 7), from a solution containing only additional citric acid; maximum—at a significantly lower pH = 4.5 (Synthesis 11) from a solution containing a group of organic acids (citric, succinic, and malic).

Even if we assume that all of those impurities located only in weddellite, replacing Ca^2+^ and did not adhere on the crystal faces, then their influence would still be too small to significantly affect the parameters of the unit cell (determined via a standard XRPD experiment). This leads to an assumption that the observed significant changes in weddellite parameters, primarily the parameter ***a*** (Appendix A Table A2), were mainly due to the changes in the amount of zeolite water. Calculated from the value of the parameter ***a***, the content of zeolite water *x* in the synthesized weddellites varied from 0.05 to 0.44 apfu. In most cases, *x* values were in the range of 0.08–0.20 apfu [21]. Weddellite with the smallest amount of water (*x* = 0.05) was synthesized in alkaline medium (pH_init_ = 8.6) from a solution containing only citric acid.

The average CSD size of nanoscale synthesized weddellites varied from ~50 to 270 Å (Appendix A Table A2).

## 3. Discussion

The results of the experimental study (Appendix A Table A1 and Table A2) made it possible to identify the chemical components of biofilms and their combinations, the presence of which led to the formation of weddellite (often together with whewellite) in the area of whewellite crystallization in a narrow interval of pH_init_ = 4.5–6.5, which characterizes the acidity of the medium during the stationary phase of micromycete growth [19].

### 3.1. The Influence of Medium Chemistry on the Formation of Weddellite and the Content of Zeolite Water in It

The maximum amount of weddellite (at least 95 wt%) formed when citric acid had been added to the system that is generally released by microscopic fungi in nature and is almost always present in biofilms. A number of ions coming from the environment (from underlying substrate and air) and their combinations (K^+^, Mg^2+^, Fe^3+^, K^+^ + Mg^2+^ + PO_4_^3−^ and K^+^ + Mg^2+^ + Fe^3+^ + SO_4_^2−^ + PO_4_^3−^) were also identified, the addition of which, together with citric acid, practically did not reduce the amount of weddellite. The content of zeolite water in the synthesized weddellites varied within the range *x* = 0.08–0.39.

In most cases, the addition of organic acids and environmental ions (in different ratios) to citric acid reduces the amount of weddellite to varying degrees.

Weddellite also prevailed over whewellite (the content of weddellite was more than 50 wt% and less than 95 wt%) in the presence of the following additives in the system in addition to citric acid: malic acid, K^+^ + PO_4_^3−^, Mg^2+^ + SO_4_^2−^, Mg^2+^ + CO_3_^2−^, Fe^3+^ + SO_4_^2−^, K^+^+Mg^2+^+ PO_4_^3−^, K^+^ + Mg^2+^ + SO_4_^2−^ + PO_4_^3−^). The content of zeolite water in the synthesized weddellites varied within the range *x* = 0.15–0.31.

In natural biofilms with a predominance of crustose lichens, whewellite prevails over weddellite [8,9]. In the presented syntheses, whewellite prevailed over weddellite (the content of weddellite was more than 5 wt%, but less than or equal to 50 wt%) when either of the following additives was added to citric acid:

- Organic acids (fumaric + malic, succinic + malic, fumaric + succinic + malic);
- Environmental ions (CO_3_^2−^, Mg^2+^ + SO_4_^2−^ + CO_3_^2−^, K^+^ + PO_4_^3−^ + Sr^2+^, K^+^ + Mg^2+^ + Fe^2+^ + SO_4_^2−^ + PO_4_^3−^ + CO_3_^2−^);
- Environmental ions (K^+^ + Mg^2+^ + Fe^2+^ + SO_4_^2−^ + PO_4_^3−^ + CO_3_^2−^) simultaneously with either of the combinations of organic acids: fumaric, succinic, malic, fumaric + succinic, malic + fumaric, malic + succinic; or malic + succinic + fumaric.

Weddellite (with *x* = 0.25) in an amount of 12 wt% was obtained in the synthesis without citric acid, in the presence of the following combination of organic acids: fumaric + succinic + malic in solution. 

The content of zeolite water *x* in the weddellites synthesized in these systems varied from 0.20 to 0.44.

Crystallization of practically monophasic whewellite (the content of weddellite did not exceed 3 wt%) occurred with the addition of only environmental ions in any combinations (in the absence of organic acids in the solution).

In general, the results of the presented experiments have shown that biofilm medium chemistry has a significant effect on the crystallization of weddellite in the whewellite stability field. Minor changes in the composition of impurity components can lead to significant changes in the weddellite/whewellite ratio. The effect of the combination of additives on this ratio does not obey the law of additivity. The content of weddellite in the systems containing a representative set of both organic acids and environmental ions is no more than 23 wt%, which correlates well with natural systems.

The content of zeolite water *x* in the synthesized weddellites varied from 0.08 to 0.44 apfu, which includes the range that characterizes weddellites of biofilms *x* = 0.24–0.36 (calculated based on weddellite unit cell parameters in [25]). It should be borne in mind that, since *x* is calculated from the parameter ***a*** of the unit cell neglecting the possible effects of micro-isomorphism on it, the *x* values can be slightly overestimated. On the example of human kidney stones, it was shown that *x* values in the range 0.1–0.2 apfu characterize relatively stable weddellites [21]. The variability of the *x* value of weddellites synthesized by us indicates their different stability. We obtained relatively stable weddellites from solutions with the presence of both organic acids, primarily citric acid, and environmental ions in combination with organic acids.

### 3.2. The Influence of pH on Weddellite Formation and Its Characteristic

It was shown by us earlier [23] that, with variations in the initial pH values of the solution, and with the addition of only citrate ions, whewellite and weddellite formed. The ratio between them changed from pure whewellite to monophasic weddellite, with an increase in pH from 1.8 to 9.5. Namely, at pH = 1.8–3.8, only whewellite crystallized; at pH = 3.8–4.5, a mixture of weddellite and whewellite formed; and at pH = 4.5–9.5, only weddellite. In the syntheses carried out in this work, with the addition of citric acid at pH values ranging from 5.0 to 8.6 (Appendix A Table A1, Syntheses 4–7), monophasic weddellite formed. The content of zeolite water *x* decreased from 0.22 to 0.05 with an increase in the pH of the crystallization medium.

In the precipitates obtained from solutions with the addition of citrate ions and Fe^3+^ cations, the regularities of the formation of calcium oxalates are similar, but the formation of whewellite together with weddellite occurred in the range of pH = 3.7–5.0 [23]. For syntheses carried out at pH = 4.0–6.7 (Appendix A Table A1, Syntheses 16–19), the formation of a mixture of whewellite and weddellite at pH = 4.0, and pure weddellite at high pH values (5.0–6.1), are also common. The content of zeolite water *x* also decreased (from 0.28 to 0.08) as the pH of the crystallization medium increased.

In all the syntheses described in this section (in solutions of the same composition but with varying pH), the CSD values at first increased with an increase of pH (from ~70–100 Å to ~160 Å), and then decreased (to ~70 Å). Thus, each system had a pH value for which the CSD is maximum.

The results of the described experiments showed that the pH of the medium influences the weddellite/whewellite ratio, as well as the CSD size of the synthesized weddellites.

### 3.3. The Influence of Biofilm Additives on the Morphology of Calcium Oxalates

Weddellite observed in biofilms is characterized by a dipyramidal and dipyramidal-prismatic habit [8,9]. The present experiments made it possible to identify the solution additives that significantly affect the morphology of weddellite crystals. The synthesized weddellite crystals can be divided into three main groups by their morphology:

1. Single dipyramidal or dipyramidal–prismatic crystals.
2. Skeletal crystals with a flattened dipyramid.
3. Regular intergrowths.

The chemical composition of the crystallization medium significantly affects the morphology of the formed calcium oxalates (Figure 4, Figure 5 and Figure 6). The appearance of the faces of a tetragonal prism occurred due to the presence of citric acid and the following ions: K^+^, Sr^2+^, Mg^2+^ + SO_4_^2−^ and CO_3_^2−^ (Figure 5). The greatest development of prism faces occurs with the addition of Sr^2+^ ions. Most likely, these impurities’ combinations, or complexes which they form with solution components, are able to adsorb on the faces of the weddellite prism, leading to a general inhibition of the growth rate of these faces.

Skeletal crystals with quadrangular flattened dipyramid, in most cases, are the result of additives of organic acids, which are present along with environmental ions in the solution (Figure 4e). In systems with citrate ions, not only dipyramidal crystals, but also crystals in the form of flattened square plates with a small dipyramid in the center of the plate precipitated (Figure 4b). This is an example of the skeletal growth of such crystals under conditions of high initial supersaturation, due to the excess of the concentration of calcium cations relative to the concentration of oxalate ions. When other organic acids had been added to the system with citrate ions, weddellite crystallized in the form of strongly flattened square plates (Figure 4c). During the crystallization of weddellite with citrate ions and various inorganic additives, dipyramidal crystals changed their morphology depending on the type of the additive. The presence of magnesium cations, carbonate ions, or iron and sulfate ions in the solution promoted the crystallization of weddellite not in the dipyramidal form, but in the form of flattened quadrangular plates with dipyramidal turrets protruding in the central part of the crystal (Figure 4c–e). Iron cations promoted the formation of lamellar crystals with a wavy surface and pyramidal faces in the center (Figure 4e), on the surface of which wavy grooves and rows of dark dots were visible, which are characteristic of partial poisoning of the growing surface by islands of large complexes or accompanying crystal phases that prevent the propagation of growth layers along the face and ultimately form an uneven surface.

Different regular weddellite intergrowths (for example, with a rotation around the axis of symmetry of the fourth order or accretion along one of the planes of symmetry of sub-individuals) most often formed in the presence medium of a combination of inorganic components and organic acids in the crystallization (Figure 6d). Twin aggregates of two main types were also observed: eight-folds formed by the intergrowth of two quadrangular plates having a common fourth-order symmetry axis and unfolded around it relative to each other by 45° (Figure 6d), and various intergrowths of two or three flattened dipyramidal crystals of weddellite along a common plane of symmetry (Figure 6b,c).

Whewellite in biofilms was represented by numerous small poorly defined elongated lamellar crystals [8,9]. The results of the experiments showed that, in the presence of inorganic additives, whewellite plates decreased in size and often formed branched intergrowths consisting of lamellar plates (Figure 3). The addition of organic acid anions to the system contributed to the bending of whewellite plates, grouped into spherulites or rounded kidney-shaped branched aggregates.

### 3.4. Mechanisms of the Interaction of Additives with Crystallizing Calcium Oxalates

The solution additives actively interact with crystallizing oxalates, adsorbing on the faces of growing crystals and/or replacing Ca^2+^ ions [3]. Variations in the morphology of calcium oxalate crystals synthesized from solutions of various compositions are well explained by selective adsorption of the admixture components (organic products of fungal metabolism, primarily citric acid, several environmental ions, etc.) to the growing individual. For example, as shown above, the differently developed prism faces of weddellite are well explained by the preferential adsorption of specific admixture components on these faces. Variations in the composition of impurities adsorbed on the prism faces lead to various degrees of inhibition of its growth and development (Sr^2+^ ions give the maximum observed effect). It is also likely that strong adsorption of impurities with oxalate crystals during their growth led to their intensive splitting and the formation of various regular and irregular intergrowths, as well as skeletal forms that are not found in natural biofilms. This distinction between the morphology of natural and synthetic crystals is probably due to the fact that the ionic supersaturation of the solutions and the crystal growth speed under the natural conditions are not as high as in experiments. Replacement of calcium ions in whewellite and weddellite by divalent cations (Sr^2+^ and Mg^2+^) from the solution is also possible. The patterns of strontium incorporation into whewellite and weddellite was studied by us earlier [24]. An uneven distribution of isomorphic impurities near the surface of a growing face can cause local internal stress, which distorts the surface structure and inhibit the growth rate of the face [26].

The decrease in the size of weddellite crystals is most likely associated with an increase of nucleation centers amount of these crystals during the stage of their spontaneous crystallization. A large number of impurity ions can serve as the crystallization centers. An increase in the size of weddellite crystals is most likely due to a decrease in the number of centers of nucleation of weddellite, as well as with the specific effect of anions of various organic acids present in the medium. We can also assume that several additives are able to inhibit the nucleation of whewellite crystals in solution, while weddellite crystals nucleate and grow.

It can also be assumed that a change in the chemistry of the crystallization medium shifts the boundaries of weddellite formation. As shown by Novosel et al [27], after a specific change in the concentrations of the main components of the crystallization medium and pH, weddellite began to crystallize first, while whewellite precipitated only after some time. To further study the thermodynamic conditions of calcium oxalates formation, additional special experiments are required.

## 4. Materials and Methods

### 4.1. Synthesis

Calcium oxalates (weddellite and whewellite) were synthesized by draining solutions of anhydrous calcium chloride (CaCl_2_, 99%, Vekton, Rochester, NY, USA) and sodium oxalate (Na_2_C_2_O_4_, 99%, Vekton) in a volume of an aqueous solution (0.5 L, de-ionized water) for 3–5 days, at a temperature of 22–25 °C (which corresponds temperate environmental conditions), in weak acidic medium (pH = 4.5–6.5, Appendix A Table A1), which characterizes the acidity of the medium during the stationary phase of micromycete growth [19]. Synthesis 7, to assess the effect of the medium pH on the formation of weddellite in the presence of citric acid, was carried out in an alkaline medium (pH = 8.6–7.5). The pH value of the initial solutions (pH_init_) was set by using small additions of the sodium hydroxide (NaOH, 99%, Vekton) solution, taking into account the different buffer capacities of the solutions and the effect on the buffer properties of various impurities for each individual solution. After complete precipitation, the pH of the solution in equilibrium with the precipitate (pH_fin_) was measured. The value of the ratio between the concentration of calcium cations and oxalate ions was set in the narrow range, from 4.0 to 5.5, since, as was shown by us earlier, such a non-stoichiometric ratio of components contributes to the formation of a significant amount of weddellite [23]. Over time, the value of this ratio increased significantly due to the formation of oxalate precipitate. The various additives, characteristic of biofilms on the surface of stone monuments and buildings in urban environment, were added to the solution. Organic acids excreted by micromycetes included the following: citric (H_8_C_6_O_7_·H_2_O, 99%, Vekton), fumaric (H_4_C_4_O_4_, 99%, Vekton), malic (H_6_C_4_O_5_, 99%, Vekton) and succinic (H_6_C_4_O_4_, 99%, Vekton). Environmental ions, which come from atmospheric air, soil and underlying stone substrate, included the following: K^+^ (potassium chloride, KCl, 99%, Vekton), Mg^2+^ (magnesium chloride hexahydrate, MgCl·6H_2_O, 98%, Vekton, magnesium sulfate heptahydrate, MgSO_4_·7H_2_O, 99%, Vekton), Sr^2+^ (strontium nitrate, Sr(NO_3)2_, 99%, Vekton), Fe^3+^ (iron chloride hexahydrate, FeCl_3_·6H_2_O, 98%, Vekton), SO_4_^2−^ (magnesium sulfate heptahydrate, MgSO_4_·7H_2_O, 99%, Vekton, sodium sulfate anhydrous, Na_2_SO_4_, 98% Vekton), PO_4_^3−^ (monopotassium phosphate KH_2_PO_4_, 98%, Vekton) and CO_3_^3−^ (sodium carbonate, Na_2_CO_3_, 99%, Vekton). The contents of organic acids varied from 0.08 to 4.0 mmol/L, and were comparable to or slightly exceeded the actual concentrations of acids due to the acid-forming activity of micromycetes [28]. The concentration of monovalent cations, which were added in the form of chlorides, usually varied from 10 to 30 mmol/L. In some syntheses, the concentration of potassium cations reached 63 mmol/L. The concentrations of di- and trivalent cations were set as follows: Fe^3+^—0.04 mmol/L, Mg^2+^—2.0 mmol/L and Sr^2+^—1.0 or 2.0 mmol/L. The concentrations of various anions were sulfate ions—2.0 mmol/L, phosphate ions—3.0 mmol/L and carbonate ions—5.0 mmol/L.

The resulting precipitate was filtered, washed with distilled water and dried at room temperature. The pH of the solutions was measured by using a 410 Basic pH meter with a standard error.

### 4.2. Methods

#### 4.2.1. X-ray Powder Diffraction (XRPD)

The method was used to determine the phase composition of the precipitates. The measurements were performed by using a D2 Phaser (Bruker, Karlsruhe, Germany) powder diffractometer (CuKα radiation of wavelength λ = 1.54178 Å). X-ray diffraction patterns were collected at room temperature, in the range of 5–60° 2θ, with a step of 0.02° 2θ. Phase identification was carried out by using the ICDD PDF-2 Database (release 2016). The unit cell parameters and the average size of coherently scattering domains (CSD) were refined by using TOPAS 4.2 (Bruker, Karlsruhe, Germany) software package [29]. The calculation of the amount of zeolite water *x* in weddellites was carried out according to the value of the parameter a of the unit cell, using the linear regression equation *x* = 5.43***a*** − 66.8 [21].

#### 4.2.2. Scanning Electron Microscopy (SEM) and Energy-Dispersive X-ray (EDXS) Spectroscopy

SEM was used for the analysis of morphology of calcium oxalate crystals. Tetragonal weddellite and monoclinic whewellite crystals and their intergrowths were identified on SEM images by their previously described morphological features [30].

The distribution and qualitative elemental composition of mineral grains in biodeposits were determined via SEM and EDXS methods, respectively. Measurements were performed by means of the Desktop Scanning Electron Microscope TM3000 (Hitachi, Tokyo, Japan), which was equipped with energy-dispersive microanalysis attachment and Everhart-Thornley secondary electron detector (Oxford Instruments plc, Abingdon, UK) based on the highly sensitive YAG crystal with the resolution of 0.1 Z of the atomic number, as well as a Zeiss Supra 40 VP (Carl Zeiss AG, Oberkochen, Germany) electron microscope equipped with a variable-pressure secondary electron (VPSE) detector at an accelerating voltage of 2 or 5 kV (depending on the image resolution). The specimens were coated with carbon (~15 nm). Magnification range varied from 100× to 1000×. The EDX spectra were analyzed by means of the EDAX Genesis (AMETEK, Inc., Berwyn, PA, USA) software package (semi-quantitative analysis was performed by using a standard-less method that is generally reliable for elements with Z > 0).

## 5. Conclusions

The experimental study showed that the changes in the chemical composition of natural biofilms, which are open systems, can significantly influence the crystallization of calcium oxalates occurring under the action of fungi and lichens.

It was revealed that organic acids (citric, malic, succinic and fumaric) secreted by microscopic fungi, as well as several ions (K^+^, Mg^2+^, Fe^3+^, Sr^2+^, SO_4_^2−^, PO_4_^3−^ and CO_3_^2−^), or their combinations, coming from the environment, significantly affect the weddellite/whewellite ratio and the morphology of these phases. The appearance of the faces of a tetragonal prism, characteristic of weddellite in biofilms, according to our data, occurs due to the presence of citric acid and the following additives: K^+^, Sr^2+^, CO_3_^2−^ and Mg^2+^ + SO_4_^2−^.

The strongest unique effect leading to intensive monophasic weddellite crystallization is caused by the presence of citric acid in the medium. Minor changes in the composition of the additive components can lead to significant changes in the weddellite/whewellite ratio. The effect of the combination of the additives on this ratio does not obey the law of additivity. The content of weddellite in the systems containing a representative set of both organic acids and environmental ions is ~20 wt%, which correlates well with natural systems.

The obtained results contribute to the study of the patterns of microbial biomineralization and the development of nature-like technologies, using the metabolism of microscopic fungi.

## Data Availability

All raw data are available from corresponding authors under request.

## Appendix A

**Table A1:** Conditions of calcium oxalates syntheses by chemical deposition (temperature 21–25 °C, Ca^2+^/C_2_O_4_^2−^ = 4–5).

Synthesis No.	Additives in Solution *		pH	
	Organic Acids	Environmental Ions	Initial	Final
Without additives				
1	-	-	6.36	6.46
Environmental ions only				
2	-	K^+^, Mg^2+^, SO_4_^2−^	6.82	6.79
3	-	K^+^, Mg^2+^, Fe^3+^, SO_4_^2−^, PO_4_^3−^	5.02	4.94
Organic acids only				
4	citric	-	5.01	5.35
5	citric	-	5.56	6.20
6	citric	-	6.06	6.90
7	citric	-	8.60	7.48
8	citric, malic	-	5.25	5.47
9	citric, fumaric, malic	-	4.52	4.62
10	citric, fumaric, succinic, malic	-	4.46	4.57
11	citric, succinic, malic	-	4.45	4.56
12	fumaric, succinic, malic	-	5.82	6.17
Citric acid + environmental ions				
13	citric	K^+^	5.82	5.62
14	citric	Mg^2+^	4.32	4.90
15	citric	Mg^2+^	6.5	5.61
16	citric	Fe^3+^	4.01	4.60
17	citric	Fe^3+^	5.00	5.22
18	citric	Fe^3+^	5.48	5.88
19	citric	Fe^3+^	6.07	7.01
20	citric	CO_3_^2−^	4.78	5.34
21	citric	K^+^, PO_4_^3−^	4.18	4.82
22	citric	Mg^2+^, SO_4_^2−^	5.06	5.36
23	citric	Mg^2+^, CO_3_^2−^	6.00	5.15
24	citric	Fe^3+^, SO_4_^2−^	4.62	4.72
25	citric	K^+^, Mg^2+^, PO_4_^3−^	4.78	5.32
26	citric	Mg^2+^, SO_4_^2−^, CO_3_^2−^	5.72	6.46
27	citric	K^+^, Mg^2+^, PO_4_^3−^	6.50	5.01
28	citric	K^+^, Sr^2+^, PO_4_^3−^	6.50	6.81
29	citric	K^+^, Mg^2+^, SO_4_^2−^, PO_4_^3−^	4.90	6.75
30	citric	K^+^, Mg^2+^, Fe^3+^, SO_4_^2−^, PO_4_^3−^	4.73	6.45
31	citric	K^+^, Mg^2+^, Fe^2+^, SO_4_^2−^, PO_4_^3−^, CO_3_^2−^	4.91	5.62
Organic acid combinations and K^+^, Mg^2+^, Fe^3+^, SO_4_^2−^, PO_4_^3−^ ions				
32	citric, fumaric	K^+^, Mg^2+^, Fe^3+^, SO_4_^2−^, PO_4_^3−^	4.66	5.09
33	citric, succinic	K^+^, Mg^2+^, Fe^3+^, SO_4_^2−^, PO_4_^3−^	4.64	5.01
34	citric, malic	K^+^, Mg^2+^, Fe^3+^, SO_4_^2−^, PO_4_^3−^	4.87	5.84
35	citric, fumaric, succinic	K^+^, Mg^2+^, Fe^3+^, SO_4_^2−^, PO_4_^3−^	4.78	5.21
36	citric, malic, fumaric	K^+^, Mg^2+^, Fe^3+^, SO_4_^2−^, PO_4_^3−^	4.69	5.17
37	citric, malic, succinic	K^+^, Mg^2+^, Fe^3+^, SO_4_^2−^, PO_4_^3−^	4.72	5.20
38	citric, malic, fumaric, succinic	K^+^, Mg^2+^, Fe^3+^, SO_4_^2−^, PO_4_^3−^	4.67	5.21

* Na^+^ и Cl^−^ ions were present in all solutions.

**Table A2:** The content of weddellite in the precipitate obtained via XRPD, unit cell parameters of weddellite and the content of zeolite water in it (*x*).

Synthesis No. (Table A1)	Wd Content via XRPD, wt%	Weddellite Characterization			
		*a*, Å	*c*, Å	CSD, Å	*x*, apfu *
Without additives					
1	0	-	-	-	-
Environmental ions only					
2	0	-	-	-	-
3	3	no data	no data	no data	no data
Organic acids only					
4	100	12.342(1)	7.352(1)	104(1)	0.22
5	100	12.374(1)	7.359(1)	107(2)	0.39
6	100	12.339(1)	7.359(1)	157(3)	0.20
7	100	12.312(2)	7.343(1)	72(1)	0.05
8	75	12.329(1)	7.352(1)	75(1)	0.15
9	39	12.346(1)	7.352(1)	218(10)	0.24
10	23	12.359(1)	7.357(1)	204(15)	0.31
11	24	12.383(2)	7.362(1)	168(13)	0.44
12	12	12.35(1)	7.357(8)	55(4)	0.24
Citric acid + environmental ions					
13	99	12.338(1)	7.352(1)	98(1)	0.20
14	80	12.345(1)	7.353(1)	196(5)	0.23
15	100	12.370(2)	7.363(1)	122(2)	0.37
16	41	12.353(5)	7.358(3)	72(2)	0.28
17	100	12.348(1)	7.353(1)	158(2)	0.25
18	100	12.334(1)	7.350(1)	140(2)	0.17
19	100	12.317(3)	7.348(2)	70(1)	0.08
20	23	12.351(3)	7.357(2)	115(6)	0.27
21	76	12.358(1)	7.353(1)	195(4)	0.30
22	61	12.348(1)	7.355(1)	131(3)	0.25
23	88	12.351(1)	7.356(1)	267(7)	0.27
24	63	12.360(1)	7.355(1)	186(6)	0.31
25	62	12.349(1)	7.352(1)	188(5)	0.26
26	27	12.367(6)	7.357(3)	76(6)	0.35
27	98	12.350(1)	7.357(1)	202(4)	0.26
28	22	12.376(1)	7.368(1)	148(6)	0.40
29	89	12.356(1)	7.354(1)	118(2)	0.29
30	100	12.354(1)	7.353(1)	66(1)	0.28
31	9	12.367(11)	7.366(6)	77(8)	0.35
Organic acid combinations and K^+^, Mg^2+^, Fe^3+^, SO_4_^2−^ and PO_4_^3−^ ions					
32	17	12.354(4)	7.356(2)	128(9)	0.28
33	15	12.362(4)	7.356(2)	158(19)	0.33
34	20	12.339(1)	7.530(1)	207(14)	0.20
35	21	12.341(9)	7.352(5)	75(4)	0.21
36	9	12.376(16)	7.366(9)	52(5)	0.40
37	16	12.348(8)	7.358(4)	87(6)	0.25
38	23	12.352(3)	7.356(2)	121(6)	0.27

* Calculated by using the value of the parameter ***a*** via the linear regression equation [21].

**Table A3:** Elemental composition of precipitates, wt% (EDXS data) *.

No. (Table A1 and Table A2)	Ca	Mg	Fe	Sr	K	Na	Σ Cations	P	S	Cl
1	32.4	0.0	0.0	0.0	0.0	0.1	0.1	0.0	0.0	0.1
2	30.9	0.0	0.0	0.0	0.0	0.1	0.1	0.0	0.2	0.1
3	36.7	0.0	1.7	0.0	0.2	0.1	2.0	1.3	0.2	0.1
4	37.3	0.0	0.0	0.0	0.0	0.1	0.1	0.0	0.0	0.1
13	27.5	0.0	0.0	0.0	0.4	0.1	0.5	0.0	0.0	0.1
14	33.6	0.0	0.0	0.0	0.0	0.1	0.1	0.0	0.0	0.1
15	22.7	0.1	0.0	0.0	0.0	0.1	0.2	0.0	0.0	0.1
16	34.9	0.0	0.2	0.0	0.0	0.1	0.3	0.0	0.0	0.1
17	41.3	0.0	0.4	0.0	0.0	0.2	0.6	0.0	0.0	0.1
18	29.7	0.0	0.2	0.0	0.0	0.0	0.2	0.0	0.0	0.1
19	41.0	0.0	0.3	0.0	0.0	0.2	0.5	0.0	0.0	0.1
21	34.8	0.0	0.0	0.0	0.1	0.1	0.2	0.0	0.0	0.1
22	40.7	0.0	0.0	0.0	0.0	0.1	0.1	0.0	0.1	0.1
23	19.5	0.0	0.0	0.0	0.0	0.2	0.2	0.0	0.0	0.1
24	35.1	0.0	0.3	0.0	0.0	0.1	0.4	0.0	0.1	0.1
25	33.5	0.0	0.0	0.0	0.1	0.1	0.2	0.1	0.0	0.1
27	24.9	0.1	0.0	0.0	0.0	0.2	0.3	0.0	0.0	0.1
28	22.5	0.0	0.0	2.1	0.0	0.1	2.3	0.0	0.0	0.1
29	34.9	0.1	0.0	0.0	0.4	0.3	0.8	3.0	1.4	0.1
30	31.2	0.2	0.3	0.0	0.4	0.4	1.3	2.8	1.5	0.1
32	44.0	0.0	0.1	0.0	0.1	0.1	0.3	0.1	0.1	0.1
33	48.5	0.1	0.1	0.0	0.0	0.1	0.3	0.1	0.1	0.1
34	35.9	0.1	0.1	0.0	0.0	0.1	0.3	0.1	0.1	0.1
35	38.3	0.0	0.1	0.0	0.0	0.1	0.2	0.1	0.1	0.1
36	33.0	0.0	0.1	0.0	0.0	0.1	0.2	0.1	0.1	0.1
37	36.8	0.0	0.1	0.0	0.0	0.1	0.2	0.1	0.1	0.1
38	31.5	0.0	0.1	0.0	0.0	0.1	0.2	0.1	0.1	0.1

* Standard deviation ± 1 wt%.
